# Mesenchymal Stem Cell-Derived Exosomes Enhance 3D-Printed Scaffold Functions and Promote Alveolar Bone Defect Repair by Enhancing Angiogenesis

**DOI:** 10.3390/jpm13020180

**Published:** 2023-01-19

**Authors:** Xiaodi Sun, Yupu Mao, Beibei Liu, Ke Gu, Han Liu, Wei Du, Ruixin Li, Jian Zhang

**Affiliations:** 1 Tianjin Stomatological Hospital, Tianjin Key Laboratory of Oral and Maxillofacial Function Reconstruction, Tianjin 300041, China; 2 The Affiliated Stomatological Hospital of Nankai University, School of Medicine, Nankai University, Tianjin 300071, China; 3 Beijing Friendship Hospital, Capital Medical University, Beijing 100050, China; 4 Tianjin Key Laboratory of Blood Cell Therapy Technology, Tianjin 300384, China; 5 Union Stem Cell & Gene Engineering Co., Ltd., Tianjin 300384, China

**Keywords:** three-dimensional-printed scaffold, exosome, bone tissue engineering, bone regeneration

## Abstract

The reconstruction of severe alveolar bone defects remains a complex and challenging field for clinicians. Three-dimensional-printed scaffolds can adapt precisely to the complicated shape of the bone defects, which is an alternative solution to bone tissue engineering. Our previous study constructed an innovative low-temperature 3D-printed silk fibroin/collagen I/nano-hydroxyapatite (SF/COL-I/nHA) composite scaffold with a stable structure and remarkable biocompatibility. However, the clinical translation of most scaffolds is limited by insufficient angiogenesis and osteogenesis. In this study, we investigated the effects of human umbilical cord mesenchymal-stem-cell-derived exosomes (hUCMSC-Exos) on bone regeneration, especially from the perspective of inducing angiogenesis. HUCMSC-Exos were isolated and characterized. In vitro, the effect of hUCMSC-Exos on the proliferation, migration, and tube formation of human umbilical vein endothelial cells (HUVECs) was examined. Moreover, the loading and release of hUCMSC-Exos on 3D-printed SF/COL-I/nHA scaffolds were evaluated. In vivo, hUCMSC-Exos and 3D-printed SF/COL-I/nHA scaffolds were implanted into alveolar bone defects, and bone regeneration and angiogenesis were investigated by micro-CT, HE staining, Masson staining, and immunohistochemical analysis. The results showed that hUCMSC-Exos stimulated HUVEC proliferation, migration, and tube formation in vitro, and the effect increased with increasing exosome concentrations. In vivo, the combination of hUCMSC-Exos and 3D-printed SF/COL-I/nHA scaffolds promoted alveolar bone defect repair by enhancing angiogenesis and osteogenesis. We constructed an elaborate cell-free bone-tissue-engineering system by combining hUCMSC-Exos with 3D-printed SF/COL-I/nHA scaffolds, potentially providing new ideas for treating alveolar bone defects.

## 1. Introduction

The reconstruction of severe alveolar bone defects remains a complex and challenging field for clinicians [1]. There are various causes of alveolar bone defects, including trauma, infection, congenital diseases (e.g., ectodermal dysplasia), and dental surgical interventions [2,3]. Guided bone regeneration (GBR) is a common method, which utilizes bone substitute material particle and barrier membranes to reconstruct small alveolar bone defects. However, this procedure is inadequacy for the large bone defects due to the limitation of angiogenesis and osteogenesis [4]. Currently, the treatment strategy of autogenous bone grafting is widely used as the “gold standard” for the repair of bone defects [5]. However, this strategy has significant shortcomings that restrict its clinical application, such as limited availability and the risk of injury with additional surgery [6]. Additionally, these autogenous bone grafts cannot adapt precisely to the complicated shape of the bone defect. Three-dimensional-printed scaffolds for bone tissue engineering are an alternative solution [5]. In recent decades, 3D-printed scaffolds have received extensive attention due to their customizable shapes and three-dimensional porous structure with desirable porosity [7,8]. However, a perfect scaffold material has yet to be identified [9]. Bone is a composite material with multiple constituents, including hydroxyapatite, type I collagen, and water. Therefore, it would seem logical to create a composite scaffold including a variety of biomaterials to achieve bioactivity and structural biomimicry [5]. In our previous study, we constructed an innovative low-temperature 3D-printed silk fibroin/collagen I/nanohydroxyapatite (SF/COL-I/nHA) composite scaffold with a stable structure and remarkable biocompatibility [10,11]. Although many 3D-printed scaffolds with different materials have been studied, their clinical translation is limited by the insufficient vascularization of scaffolds, especially in the reconstruction of large alveolar bone defects [12]. The improvement of angiogenesis inside biomaterials has long been a challenge for tissue engineering scaffolds [13,14]. Osteogenesis and angiogenesis mutually support the process of bone regeneration, and the lack of vascularization is unfavorable for bone defect repair. Osteogenesis is heavily reliant on effective oxygen and nutrient diffusion, as well as cell migration and distribution. The newly formed bone tissue migrates very sluggishly from the periphery to the center of the scaffold if the scaffold has insufficient angiogenesis [7]. Therefore, 3D-printed scaffolds with rapid angiogenesis ability will be ideal bone graft materials.

Angiogenesis is crucial for the process of bone defect repair. Early vascularization provides sufficient oxygen, nutrients, and precursor cells for bone regeneration, and the degree of vascularization is positively correlated with the amount of new bone formation [15]. It has been reported that the distribution of cells is usually limited to a distance of 200 μm from the nearest capillary, which is the effective diffusion distance of oxygen and nutrients [12]. In addition, vascularization also plays an important role in regulating cell signaling molecules involved in bone regeneration [16]. Various approaches have been proposed to promote the vascularization of bone graft materials, such as the use of vascular endothelial growth factor (VEGF), fibroblast growth factor (FGF), and stem cells [7,12,17]. However, bone regeneration is regulated by multiple factors that stimulate angiogenesis and osteogenesis. It is difficult to achieve the desired bone regeneration with only one bioactive growth factor. Stem cells are also not ideal supplementary factors due to issues related to cell sources, biosafety, immunological rejection, and genetic mutations [18]. Therefore, a cell-free tissue engineering strategy that delivers multiple active factors is desired to stimulate vascular development for better bone regeneration.

Exosomes are extracellular vesicles that are released from all cells, prokaryotes, and eukaryotes and have a size range of 40–160 nm in diameter [19,20]. Exosomes derived from stem cells contain many functional proteins, miRNAs, and other bioactive contents that exhibit therapeutic potential similar to that of the original cells [21,22,23]. Additionally, exosomes are multifunctional in terms of modulating cell behaviors and activating signaling pathways compared with single proteins or gene therapy [19,21]. More importantly, exosomes themselves are not real cells, which allows them to readily overcome the limitations of cell-based therapy [22]. It was reported that mesenchymal-stem-cell (MSC)-derived exosomes function in tissue regeneration [24]. Their therapeutic functions include accelerating angiogenesis, promoting cell proliferation, and modulating immune reactivity by regulating cell signaling pathways [25,26]. Therefore, we speculated that MSC-derived exosomes can improve angiogenesis and osteogenesis on 3D-printed SF/COL-I/nHA scaffolds.

In our study, we investigated the effects of human umbilical cord mesenchymal-stem-cell-derived exosomes (hUCMSC-Exos) combined with 3D-printed SF/COL-I/nHA scaffolds on bone regeneration, especially from the perspective of inducing angiogenesis. We extracted and characterized hUCMSC-Exos and examined their angiogenic potential by the proliferation, migration, and tube formation in human umbilical vein endothelial cells (HUVECs) in vitro. We explored the loading and release of exosomes on 3D-printed SF/COL-I/nHA scaffolds. Furthermore, we investigated the effect of the combined application of hUCMSC-Exos and 3D-printed SF/COL-I/nHA scaffolds on alveolar bone regeneration in vivo by micro-CT, HE staining, Masson staining, and immunohistochemical analysis. We constructed an elaborate cell-free bone-tissue-engineering system by combining exosomes with 3D-printed SF/COL-I/nHA scaffolds, potentially providing new ideas for treating alveolar bone defects.

## 2. Materials and Methods

### 2.1. Cell Culture

HUCMSCs were acquired from Union Stem Cell & Gene Engineering Co. Ltd. (Tianjin, China). The cells were cultured in α-modified essential medium (α-MEM) (Gibco, Waltham, MA, USA) supplemented with 10% fetal bovine serum (FBS, Gibco, Waltham, MA, USA) and 10% antibiotic–antifungal agent (Gibco, USA). HUCMSCs between Passages 4 and 6 were used for subsequent experiments. HUVECs were obtained from BeNa Culture Collection Co. Ltd. (Beijing, China) and expanded in endothelial growth medium-2 (EGM-2) (Lonza, Morristown, NJ, USA). All incubations were performed at 37 °C with 5% CO_2_. All experimental procedures received approval from the Ethics Committee of Tianjin Stomatological Hospital.

### 2.2. Exosome Isolation

The method used for exosome isolation was performed as previously reported [20]. Briefly, 10% FBS α-MEM was replaced with 10% exosome-free FBS when the hUCMSCs were 80–90% confluent. After 2 days, the culture medium of hUCMSCs was collected and centrifuged at 300× *g* for 10 min and then at 2000× *g* for 10 min at 4 °C to remove the cell debris. The supernatants were centrifuged for 30 min at 10,000× *g*. The medium was then filtered through a 0.22μm sterilized filter. Subsequently, the supernatant was ultracentrifuged at 140,000× *g* for 70 min to remove large cell vesicles. Exosomes were harvested after discarding the supernatant. The resulting pellet was further purified by resuspension in PBS for subsequent use.

### 2.3. Exosome Characterization

The morphology of the exosomes was detected by transmission electron microscopy (TEM; HT7700, Hitachi, Tokyo, Japan). The particle size distribution of the exosomes and lyophilized exosomes was analyzed with a ZetaViewlaser scattering instrument (Particle Metrix, Ammersee, Germany). Western blotting was conducted to examine the exosome markers CD9, CD81, and CD63 (Abcam, Cambridge, U.K.). The exosome protein concentration was determined with a BCA Protein Assay Kit (Applygen, Beijing, China).

### 2.4. Cell Proliferation

Four different concentrations of hUCMSC-derived exosomes (0, 10, 50, and 100 μg/mL) were applied to evaluate the impact on HUVEC proliferation. In brief, HUVECs (2000 cells/well) were seeded into 96-well plates in EGM-2 (Lonza) and cocultured with different concentrations of exosomes (10, 50, and 100 μg/mL) or an equivalent volume of exosome diluent (PBS) for 48 h. The optical density (OD) at 450 nm was determined with a CCK-8 kit (Dojindo, Kumamoto, Japan).

### 2.5. Cell Migration Scratch

A scratch wound-healing assay was conducted to assess the cell migration capability. Briefly, HUVECs were seeded into 6-well plates at a density of 1 × 10^5^ cells/well and, then, grown to 100% confluency. Subsequently, the confluent cell layer was scratched using a sterile 200 mL pipette tip. After washing three times with PBS to remove loose cells, PBS or 10, 50, or 100 μg/mL exosomes was added, as described above. Images were recorded at 0 and 6 h after scratching. Migration was quantified by measuring the residual fractional wound area using the ImageJ 1.53f51 software (NIH).

### 2.6. Vessel-like Formation Assay

The impact of hUCMSC-derived exosomes on angiogenesis was evaluated by a vessel-like formation assay. HUVECs were harvested, resuspended in EGM-2 with exosomes (0, 10, 50, and 100 μg/mL), and seeded into 24-well plates coated with Matrigel (Corning, NY, USA) at a concentration of 3 × 10^4^ cells/well. After incubation for 6 h, the images of the tube formation were acquired, and the total tube length and the number of junctions were measured using the ImageJ 1.53f51 software.

### 2.7. Preparation of 3D-Printed SF/COL-I/nHA Scaffolds

In this study, porous bioresorbable composite bone scaffolds composed of SF/COL-I/nHA were fabricated with low-temperature deposition 3D-printing technology, as previously reported [10,11]. Silk fibroin was mixed with collagen I and nHA at a mass ratio of 5:4:1 and stirred for 4 h. A moderate amount of acetic acid was added to aid the mixing. The prepared composite materials were loaded into the needle tubing. During the printing process, the printing parameters and floor temperature were set up to ensure the molding. The samples were then lyophilized using a freeze-dryer (SCIENTZ-12N, Zhejiang, China) at a low temperature and crosslinked under absolute alcohol at room temperature for 24 h. After crosslinking, the scaffolds were treated with NaOH (Ph = 10), washed with deionized water, sterilized with Co60, and stored at −20 °C.

### 2.8. Lyophilization and Rehydration of Exosomes

The methods used to lyophilize exosomes have been described previously [27]. In brief, the freshly extracted exosomes were stored at −80 °C overnight. Then, the samples were lyophilized in a vacuum freeze-dryer (SCIENTZ-12N, Zhejiang, China) for 8 h. The freeze-dried exosomes were rehydrated with the original volume of PBS. The morphology, particle size distribution, and concentration of the exosomes were analyzed before lyophilization and after rehydration.

### 2.9. Exosome Incorporation into 3D-Printed Scaffolds and Release Profile

Four-millimeter-long and one-millimeter-high cuboid SF/COL-I/nHA scaffolds were freeze-dried and then immersed in 1 μg/mL of exosome solution (250 μL/per scaffold) at 4 °C overnight to enable complete loading with exosomes. In addition, the same scaffolds were immersed in PBS as a control group. These two groups of compound materials were incubated in 500 μL of PBS at 37 °C. A volume of 10 μL of PBS was collected and replaced with an equal volume of fresh PBS at predetermined time points of 1, 2, 3, 4, 5, 6, 7, and 8 days. The protein concentrations of the two groups of samples (C_SF/COL-I/nHA/Exos_ and C_SF/COL-I/nHA_) were measured with a BCA Protein Assay Kit. The amount of exosome release at each time point was calculated by the formula C_SF/COL-I/nHA/Exos-_C_SF/COL-I/nHA_, and the cumulative release rate of exosomes was obtained with the formula C_X_/C_MAX_ × 100% (C_X_: the concentration of exosomes at each time point; C_MAX_: the maximum concentration of exosomes at all time points) [28].

### 2.10. Rat Alveolar Bone Defect Model

The animal experiments were approved by the Ethics Committee of Tianjin Stomatological Hospital and followed the institutional animal handling guidelines and the rules of the National Institutes of Health (IRM-DWLL-2021192). Eight-week-old male SD rats (weighing 250 ± 10 g) were used in the animal experiments. The rats were randomly divided into 2 groups with 5 rats in each group: (1) SF/COL-I/nHA-scaffold-only (SF/COL-I/nHA group) and (2) SF/COL-I/nHA scaffold + exosomes (SF/COL-I/nHA/Exos group). An alveolar bone defect was prepared as previously described [29]. Briefly, a superficial skin incision was made through the skin along the masseter muscle tendon attachment ridge, and then, the deep fascia, masseter muscle, and periosteum were separated through blunt dissection to fully expose the proposed bone defect site. Importantly, injuries to the parotid duct, facial nerve, and inferior alveolar nerve were avoided during the operation. A 4.0 × 4.0 × 1.0 mm^3^ critical-sized defect was made on the buccal side of the posterior tooth area in the mandible [29]. Abundant saline irrigation was necessary to lower the temperature. The two groups of scaffolds were implanted into the defects. After repositioning the subcutaneous tissue, the muscle layer was sutured using the horizontal mattress method, and then, the skin was sutured with interrupted sutures to close the incision. All rats were treated with antibiotics and painkillers for three days. Specimens from each group were harvested at 6 weeks after the surgical procedures. The mandibles with defects were fixed in 4% paraformaldehyde for 48 h.

### 2.11. Microcomputed Tomography Evaluation

The specimens were subjected to micro-CT scanning (Bruker SkyScan 1276, Billerica, MA, USA; 85 kV, 200 mA, 10 μm resolution). The three-dimensional reconstruction of the sagittal and axial sections was performed using the DataViewer software (Version 1.5.2.4; Bruker, Billerica, MA, USA). The new bone volume fraction (BV/TV), trabecular number (Tb.N), trabecular thickness (Tb.Th) and trabecular spacing (Tb.Sp) values were calculated using CTAn (Version 1.17; Bruker, Billerica, MA, USA).

### 2.12. Histology, Immunohistochemistry, and Histomorphometric Analysis

After micro-CT analysis, the mandibular bones were decalcified for 2 months with 10% EDTA (pH 7.4), dehydrated, and subsequently, embedded in paraffin. Then, 5 μm-thick sections were prepared for histological analysis. Haematoxylin-eosin (H & E) and Masson staining were performed to visualize defect healing and new bone formation.

The effects of osteogenesis and angiogenesis in the defect regions were evaluated by COL1 and CD31 immunohistochemistry (IHC) staining. The primary antibodies against COL1 (Abcam, Cambridge, U.K.) and CD31 (Abcam, Cambridge, U.K.) were diluted 1:2000 and 1:500, respectively, according to the manufacturer’s instructions. Images were captured using a DS-U3 imaging system (Nikon, Konan, Japan). Briefly, the percentage of COL1-positive area was analyzed by the Image-ProPlus 6.0 software. For the analysis of CD31 staining, 6 fields of view within the regions of interest (ROIs) area could be randomly selected under high magnification, and then, the number of stained circumferential vessels was counted by the image software.

### 2.13. Statistical Analysis

Statistical analyses were performed by an independent two-tailed Student’s *t*-test or one-way ANOVA using the SPSS 20.0 software (IBM Inc., Armonk, NY, USA). All the data are presented as the mean ± the standard deviation (SD) of 3–5 experiments per group. *p*-values < 0.05 were considered statistically significant.

## 3. Results

### 3.1. Characterization of hUCMSC-Derived Exosomes

**Figure 1:**
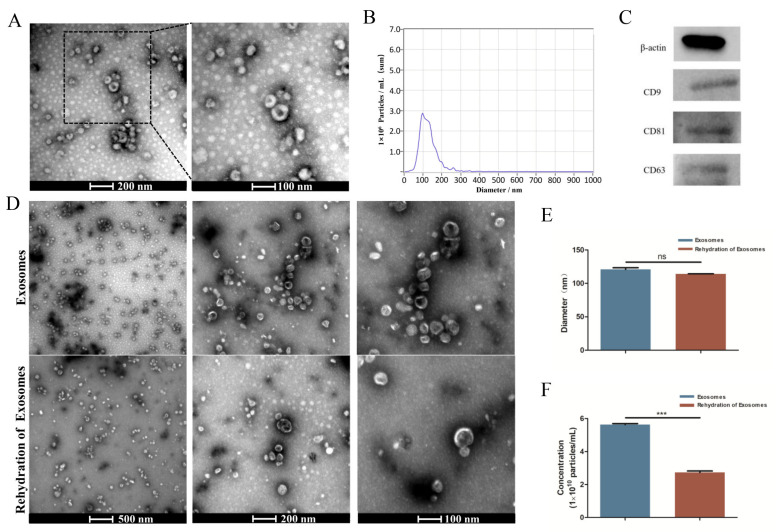
Characterization of HUCMSC-derived exosomes. (**A**) Morphology of exosomes detected by TEM. (**B**) Particle size distribution and concentration of exosomes observed by NTA. (**C**) Western blot analysis of the exosome surface markers. (**D**) Morphology of exosomes before lyophilization and after rehydration by TEM. (**E**,**F**) Size peak and concentration analysis of exosomes before lyophilization and after rehydration. ^ns^ *p* > 0.05, *** *p* < 0.001.

TEM analysis showed an obvious bilayer membrane structure and saucer-shaped morphology (Figure 1A). NTA analysis showed that the peak diameter was nearly 100 nm, and the range of the particle size had close to a normal distribution (Figure 1B). The detection results had high confidence. Western blot analysis revealed that the exosomes expressed the characteristic surface markers CD9, CD81, and CD63 (Figure 1C). All of these results suggested that the exosomes were successfully extracted. The results obtained after the rehydration of the freeze-dried exosomes showed no significant change in the saucer-shaped morphology nor diameter (Figure 1D,E), but the number of particles was reduced by nearly half (Figure 1F).

### 3.2. Effects of hUCMSC-Derived Exosomes on Proliferation, Migration, and Tube Formation in HUVECs

**Figure 2:**
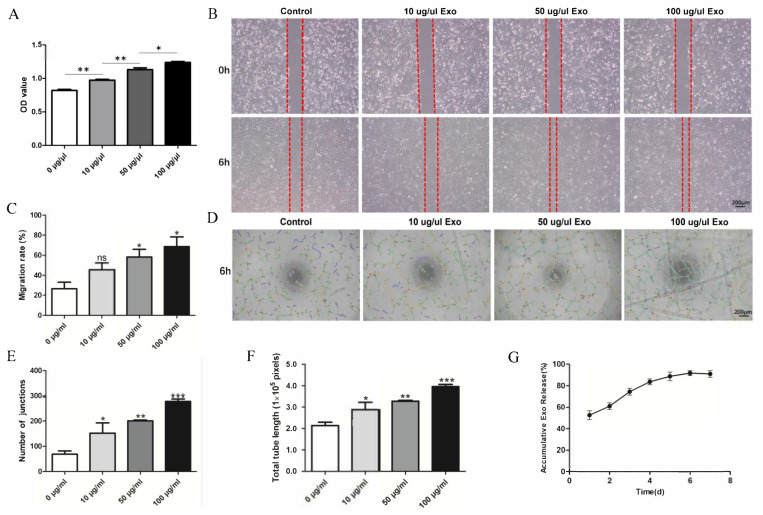
Effect of hUCMSC-derived exosomes on HUVECs. (**A**) Proliferation viability of HUVECs treated with different concentrations of exosomes. (**B**) Scratch wound-healing assay of HUVECs treated with different concentrations of exosomes. (**C**) Quantitative analysis of the scratch wound assay. (**D**) Tube formation after treatment with different concentrations of exosomes. (**E**,**F**) Quantitative evaluation of the tube length and the junction number after treating HUVECs with different concentrations of exosomes. (**G**) hUCMSC-derived exosomes’ release profile from SF/COL-I/nHA. All experiments were performed in triplicate. ^ns^ *p* > 0.05, * *p* < 0.05, ** *p* < 0.01, *** *p* < 0.001, compared with the control group.

HUVEC proliferation was evaluated by a CCK-8 assay. The OD values revealed significant differences between the groups at 24 h. This indicates that the exosomes can promote HUVEC proliferation, and the effect increased with increasing exosome concentration (Figure 2A). The migration ability of the HUVECs was significantly enhanced in a concentration-dependent manner by the exosomes (Figure 2B,C). The angiogenesis ability of HUVECs is essential for bone repair and regeneration. A tube formation assay was conducted to explore the function of hUCMSC-derived exosomes in the angiogenic differentiation of HUVECs. The data indicated that the total tube length and the number of junctions significantly increased. Compared with that of the control group, the total tube length of the exosome-treated group (100 μg/mL) was nearly four-fold greater, and the number of junctions was nearly four-fold higher (Figure 2D–F).

### 3.3. Exosome Release from SF/COL-I/nHA Scaffolds

The release profile (Figure 2G) showed that the exosomes were quickly released from the scaffolds in 0–24 h. As the incubation time increased, the cumulative release rate of the exosomes gradually increased, reaching the highest value on Day 6, after which, the cumulative release rate began to decrease.

### 3.4. Micro-CT Analysis of Bone Regeneration

**Figure 3:**
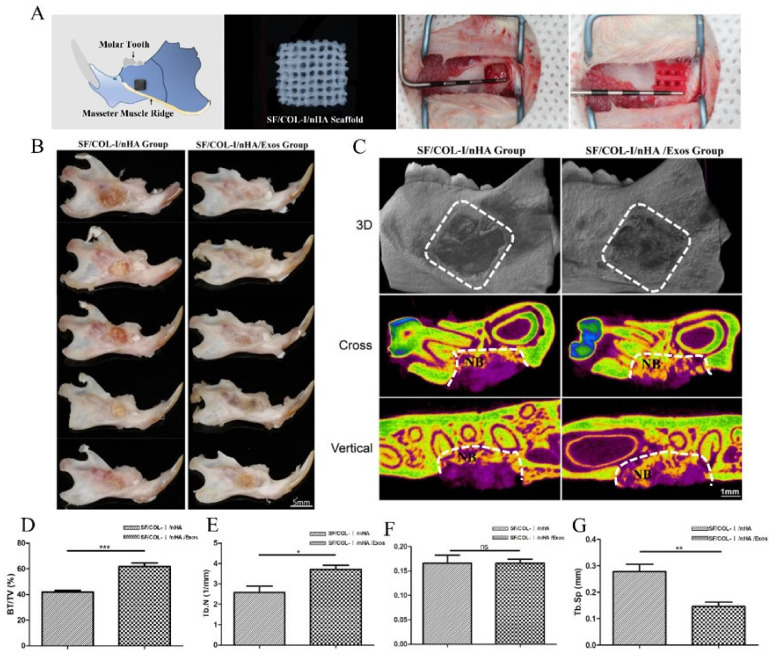
Mandibular defect, reconstruction in vivo, and micro-CT analysis of bone regeneration at 6 weeks. (**A**) Schematic diagram of bone defect model; preparation of defect model; implantation of biomaterials. (**B**) Macroscopic appearance of mandible defect following different treatments. **(C)** Reconstructed 3D micro-CT images of bone formation; 2D cross-sectional images; 2D sagittal section images. NB: new bone. (**D**) Percentage of new bone formation (BV/TV). (**E**) Trabecular number. (**F**) Trabecular thickness. (**G**) Trabecular separation. Periodontal probe with 2 mm per scale; BV: bone volume; TV: total volume; ^ns^ *p >* 0.05, * *p* < 0.05, ** *p* < 0.01, *** *p* < 0.001, compared with the control group.

Schematic diagrams of the animal models, SF/COL-I/nHA scaffolds, and surgical procedures are presented in Figure 3A. The gross specimen observation revealed obvious suppuration, and osteonecrosis was not observed in the bone defect area (Figure 3B). The boundary of the bone defect cavity in the SF/COL-I/nHA group was still relatively clear; the scaffolds were not completely degraded, and the residual structure of the scaffold was visible. In the SF/COL-I/nHA/Exos group, the area of the mandibular defect was basically healed; the bone cortex could be detected in some samples, and the scaffolds had nearly degraded and disappeared. The micro-CT results included qualitative and quantitative analyses. Three-dimensional (3D) construction showed bone regeneration in both the SF/COL-I/nHA group and the SF/COL-I/nHA/Exos group, but the bone defect boundaries of the SF/COL-I/nHA group were still clearly visible. In the two-dimensional (2D) images, the defect areas of the SF/COL-I/nHA/Exos group regenerated better than the defect areas of the group implanted with only SF/COL-I/nHA scaffolds (Figure 3C). The defect area was occupied by more new bone tissue. In accordance with the radiological images, the BV/TV ratio in the SF/COL-I/nHA/Exos group (61.86%) was higher than that in the SF/COL-I/nHA group (41.90%) in the volume of interest (VOI) region (Figure 3D). In addition, the bone quality in the VOI region was compared between the two groups. The trabecular number (Tb.N) values in the SF/COL-I/nHA/Exos group were significantly higher than those in the SF/COL-I/nHA group (Figure 3E). The trabecular thickness (Tb.Th) values were not significantly different between the two groups (Figure 3F). The trabecular separation (Th.Sp) values, which represent the average distance between the trabeculae of the bone, were significantly lower in the SF/COL-I/nHA/Exos group than in the SF/COL-I/nHA group (Figure 3G). These results indicated that the combination of SF/COL-I/nHA scaffolds and hUMSC-derived exosomes could better promote the process of bone regeneration in the defect area.

### 3.5. Histological Analysis of Bone Regeneration

**Figure 4:**
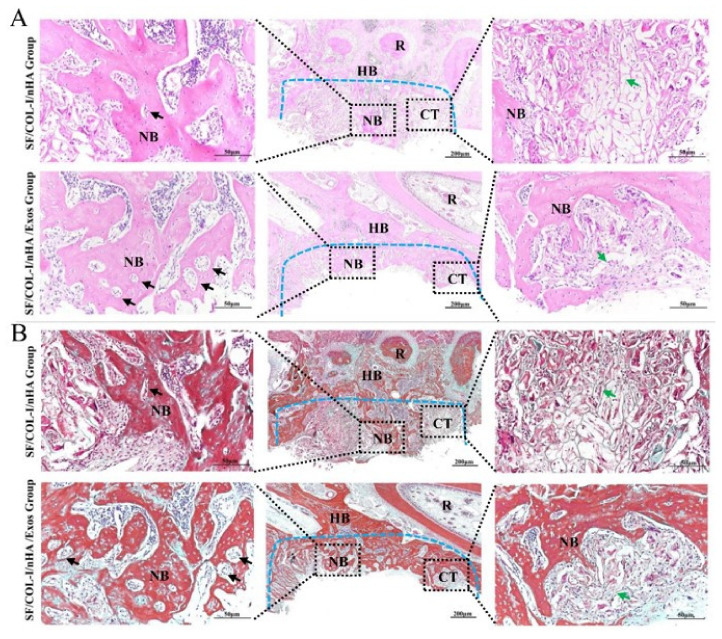
Histological results of bone regeneration at 6 weeks. (**A**) HE staining. (**B**) Masson staining. The blue dotted line means the dividing line between new bone and host bone. Black arrows indicate neovessels in the new bone. Green arrows represent the remaining SF/COL-I/nHA scaffold. NB: new bone; HB: host bone; CT: connective tissue; R: root.

The HE staining results are shown in Figure 4A. Obvious inflammatory aggregation and tissue necrosis were not observed. The bone defects of the SF/COL-I/nHA group were mainly filled with fibrotic connective tissue, and the transition zone was obvious. Compared with the SF/COL-I/nHA group, the SF/COL-I/nHA/Exos group showed increased deposition of new bone both along the border and in the center of the defects. At high magnification, a large number of mature osteocytes and new blood vessels were observed in the SF/COL-I/nHA/Exos group. The residual structure of the SF/COL-I/nHA scaffold was blurred. In contrast, a mass of fibrous tissue and obvious reticular remnants of the scaffolds were observed in the SF/COL-I/nHA group. In addition, the new bone had a low degree of mineralization and the number of new blood vessels was relatively small in the SF/COL-I/nHA group.

The Masson staining results, consistent with the HE staining results, also revealed more bone-like tissue formation in the SF/COL-I/nHA/Exos group than in the SF/COL-I/nHA group. The collagen fiber bundles in the SF/COL-I/nHA/Exos group were more mature than those in the SF/COL-I/nHA group (Figure 4B).

### 3.6. Immunohistochemical Staining for Bone Regeneration and Angiogenesis

**Figure 5:**
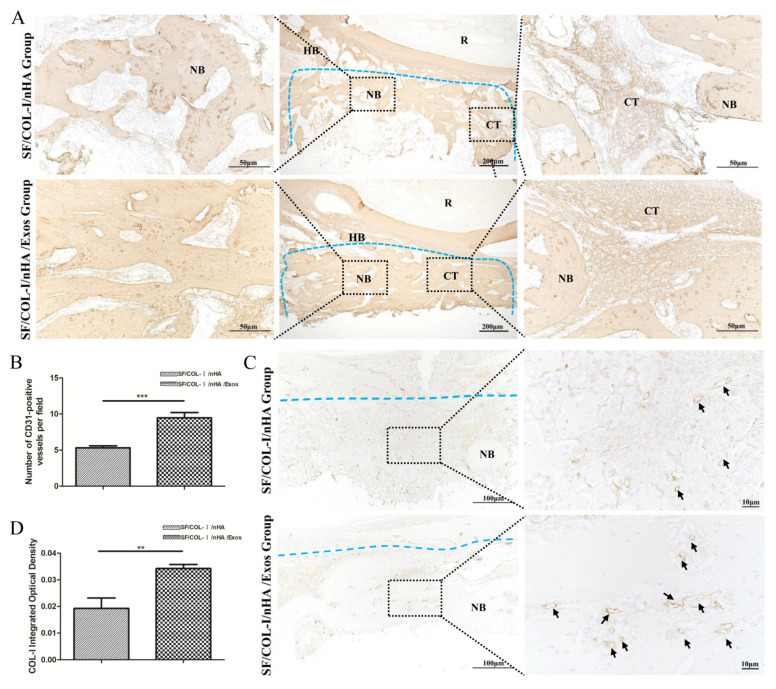
Immunohistochemical staining for bone regeneration and angiogenesis at 6 weeks. (**A**) COL1 staining. (**B**) Quantitative analysis of COL1 expression. (**C**) CD31 staining. (**D**) Quantitative analysis of CD31 expression. Brown color indicates positive expression, and the darker brown, the stronger the expression. The blue dotted line means the dividing line between new bone and host bone. Black arrows indicate blood vessels in the new bone. NB: new bone; HB: host bone; CT: connective tissue; R: root. ** *p* < 0.01, *** *p* < 0.001.

IHC staining revealed higher expression of an osteogenic marker (COL1) in the SF/COL-I/NHA/Exos group than in the SF/COL-I/nHA group (Figure 5A). Five different fields were randomly observed in the bone defect repair area, and the IHC staining intensity score values of the SF/COL-I/nHA/Exos group were significantly higher than those of the SF/COL-I/nHA group (Figure 5B). CD31 is a surface marker for vascular endothelial cells, and a large number of CD31-positive sites were observed in the SF/COL-I/nHA/Exos group compared with the SF/COL-I/nHA group (Figure 5C,D). Additionally, in the SF/COL-I/nHA/Exos group, the linear ring vascular structures had a thicker diameter, and the intensity of staining was deeper. These results suggested that more neovascularization occurred in the SF/COL-I/nHA/Exos group than in the SF/COL-I/nHA group.

## 4. Discussion

Angiogenesis plays an important role in the progression of osteogenesis, as the blood supply provides sufficient oxygen and nutrients and induces the migration of osteoblasts and the mineralization of bone tissue [30,31]. Recent studies have reported that exosomes have various potential applications in diagnosis and treatment. Exosomes have been suggested as potential regenerative agents that can replace stem cell transplantation in bone regeneration, as they deliver bioactive molecules to enhance angiogenesis and osteogenesis [32,33]. Exosomes from stem cells have the potential to be combined with bone-engineering scaffolds to repair bone defects [34]. Exosome-mediated angiogenesis has been reported. MSC-Exos can promote the proliferation, migration, and angiogenic differentiation of endothelial progenitor cells (EPCs) [35]. Mechanistic studies revealed that exosomal miR-21 promotes angiogenesis by upregulating the NOTCH1/DLL4 pathway [35]. Other reports have shown that MSC-derived exosomal miR-21 targets SPRY2 to enhance angiogenesis and exosomal miR-1260a via the inhibition of COL4A2 to promote angiogenesis [36,37]. Jing et al. reported that stem cells from apical-papilla-derived exosomes promote angiogenesis via miR-126-5p, as indicated by increased expression of VEGF and ANG-1 [38]. Additionally, Takeuchi et al. revealed that MSC-Exos promoted the expression of not only osteogenesis-related genes, but also angiogenesis-related genes such as VEGF, ANG1, and ANG2 [31]. In vivo experiments revealed that the application of exosomes secreted by human-induced pluripotent-stem-cell-derived mesenchymal stem cell (hiPSC-MSC-Exo)+β-TCP scaffolds dramatically promoted angiogenesis and bone regeneration in critical-sized calvarial defects in ovariectomized rats [39]. In this study, we further confirmed that hUCMSC-Exos had the ability to enhance HUVEC proliferation, migration, and tube formation in vitro, and the effect increased with increasing exosome concentrations. Moreover, hUCMSC-Exos+3D-printed SF/COL-I/nHA scaffolds enhanced bone regeneration and angiogenesis compared to the scaffold-only group in the context of alveolar bone reconstruction in vivo. Therefore, these results suggest that MSC-Exos can stimulate angiogenesis during bone regeneration, which shows tremendous potential for bone defect reconstruction.

The direct application of exosomes is limited by their loss and low efficiency in bone defects. Exosomes combined with the local application of biomaterials could not only reduce the number of exosomes used, which plays an important role at specific sites, but also support sufficient loading and stable delivery of exosomes. To achieve the optimal biological efficacy of exosomes, a loading and release pattern should be achieved to ensure a sufficient duration of their functions. Therefore, a carrier for exosome-based tissue engineering is needed as a loading and release system. In addition to the desirable properties of bone scaffolds, a suitable scaffold for exosome-based tissue engineering should be biocompatible, mechanically supported, and capable of loading and releasing exosomes [40]. Our previous studies proved that SF/COL-I/nHA scaffolds had ideal porosity, water absorption, biocompatibility, and mechanical properties [10,11]. In a rabbit model, the application of SF/COL-I/nHA scaffolds promoted bone regeneration compared with the blank controls, meeting the needs of tissue-engineering scaffolds [11]. The porosity and water absorption of the SF/COL-I/nHA scaffolds makes them suitable as effective carriers of drugs and growth factors [11]. However, it was not clear whether the SF/COL-I/nHA scaffolds could effectively load and release exosomes to meet the needs of exosome-based tissue engineering. In this study, 50% of the exosomes were released within 24 h, indicating burst release. Over the following 2 to 6 days, the exosomes were slowly released and gradually accumulated. Our results showed that the SF/COL-I/nHA scaffolds enabled the sustained release of the exosomes, especially during the early angiogenesis stage.

The way exosomes are loaded on scaffolds is critical for their efficiency. Our SF/COL-I/nHA scaffolds underwent a lyophilization process during production. The lyophilization technique has been used to preserve various types of biological materials, such as proteins, plasma, and living cells [27]. If exosomes can be preserved in SF/COL-I/nHA scaffolds by the freeze-drying technique, this approach will be beneficial for the preservation and transport of SF/COL-I/nHA/Exos scaffolds. However, our study showed that the morphology and particle size of the exosomes did not change significantly after freeze-drying and resolubilization, but their concentration was significantly reduced. Akers et al. also found that lyophilization caused a reduction in extracellular vesicle (EV) number [41], which is generally consistent with our results. This may be because exosomes are damaged under various stresses during the freezing and drying steps [27]. To protect exosomes from these stresses, trehalose was used as a cryoprotectant [27]. We also added trehalose in our experiments, but the concentration of exosomes after lyophilization and resolubilization did not improve significantly. Therefore, we used regular physical adsorption methods instead of freeze-drying during loading to achieve a sufficient number of exosomes in the bone defects. The development of methods for the easy preservation and transport of SF/COL-I/nHA/Exos scaffolds needs to be further explored.

## 5. Conclusions

In the present study, we demonstrated that hUCMSC-Exos effectively stimulate HUVEC proliferation, migration, and tube formation in vitro, and the effect increases with increasing exosome concentrations. Further analysis showed that 3D-printed SF/COL-I/nHA scaffolds were ideal carriers for exosome-based tissue engineering, and the application of hUCMSC-Exos+3D-printed SF/COL-I/nHA scaffolds promoted bone regeneration in alveolar bone defects through enhanced angiogenesis and osteogenesis in vivo.

## Data Availability

The data presented in this study are available upon request from the corresponding author.
